# Clinical outcomes and cumulative healthcare costs of TAVR vs. SAVR in Asia

**DOI:** 10.3389/fcvm.2022.973889

**Published:** 2022-09-21

**Authors:** Elise Chia-Hui Tan, Yung-Tsai Lee, Yu Chen Kuo, Tien-Ping Tsao, Kuo-Chen Lee, Ming-Chon Hsiung, Jeng Wei, Kuan-Chia Lin, Wei-Hsian Yin

**Affiliations:** ^1^Department of Health Service Administration, China Medical University, Taichung, Taiwan; ^2^Department of Pharmacy, Institute of Hospital and Health Care Administration, National Yang Ming Chiao Tung University, Taipei, Taiwan; ^3^Heart Center, Cheng Hsin General Hospital, Taipei, Taiwan; ^4^Faculty of Medicine, National Defense Medical Center, Taipei, Taiwan; ^5^Community Research Center, Institute of Hospital and Health Care Administration, National Yang Ming Chiao Tung University, Taipei, Taiwan; ^6^Faculty of Medicine, National Yang Ming Chiao Tung University, Taipei, Taiwan

**Keywords:** aortic stenosis (AS), transcatheter aortic valve replacement (TAVR), surgical aortic valve replacement (SAVR), real-world effectiveness, health outcomes, healthcare utilization and associated direct cost

## Abstract

**Objectives:**

This study compared transcatheter aortic valve replacement (TAVR) and surgical aortic valve replacement (SAVR) in terms of short- and long-term effectiveness.

**Methods:**

This retrospective cohort study based on nationwide National Health Insurance claims data and Cause of Death data focused on adult patients (*n* = 3,643) who received SAVR (79%) or TAVR (21%) between 2015 and 2019. Propensity score overlap weighting was applied to account for selection bias. Primary outcomes included all-cause mortality (ACM), hospitalization for heart failure, and a composite endpoint of major adverse cardiac events (MACE). Secondary outcomes included medical utilization, hospital stay, and total medical costs at index admission for the procedure and in various post-procedure periods. The Cox proportional-hazard model with competing risk was used to investigate survival and incidental health outcomes. Generalized estimation equation (GEE) models were used to estimate differences in the utilization of medical resources and overall costs.

**Results:**

After weighting, the mean age of the patients was 77.98 ± 5.86 years in the TAVR group and 77.98 ± 2.55 years in the SAVR group. More than half of the patients were female (53.94%). The incidence of negative outcomes was lower in the TAVR group than in the SAVR group, including 1-year ACM (11.39 vs. 17.98%) and 3-year ACM (15.77 vs. 23.85%). The risk of ACM was lower in the TAVR group (HR [95% CI]: 0.61 [0.44–0.84]; *P* = 0.002) as was the risk of CV death (HR [95% CI]: 0.47 [0.30–0.74]; *P* = 0.001) or MACE (HR [95% CI]: 0.66 [0.46–0.96]; *P* = 0.0274). Total medical costs were significantly higher in the TAVR group than in the SAVR in the first year after the procedure ($1,271.89 ± 4,048.36 vs. $887.20 ± 978.51; *P* = 0.0266); however, costs were similar in the second and third years after the procedure. The cumulative total medical costs after the procedure were significantly higher in the TAVR group than in the SAVR group (adjusted difference: $420.49 ± 176.48; *P* = 0.0172).

**Conclusion:**

In this real-world cohort of patients with aortic stenosis, TAVR proved superior to SAVR in terms of clinical outcomes and survival with comparable medical utilization after the procedure.

## Introduction

Aortic stenosis (AS) is associated with a high risk of death; however, many patients cannot undergo surgical aortic valve replacement (SAVR) due to time constraints and surgery-related risks. Transcatheter aortic valve replacement (TAVR) is an alternative to SAVR for patients with severe AS. Since its introduction in 2002, more than 300,000 TAVR procedures have been performed worldwide.

Some previous randomized controlled trials comparing TAVR with SAVR reported that TAVR provides significant survival benefits for high-risk patients with severe AS (1–5). Other trials in intermediate-risk patients with severe AS reported that SAVR and TAVR are similar in terms of the risk of death or disabling stroke (6–8). Recent trials in low-risk patients reported that TAVR is superior to SAVR with respect to the composite rate of all-cause mortality (ACM), stroke or rehospitalization in the first year after the procedure (9), and non-inferior in the second year (10). Similar non-inferior findings have been reported in other clinical trials of low-risk patients (11, 12). Previous studies have also reported that pacemaker use and the incidence of left bundle branch block (LBBB) were higher among patients who received TAVR (11, 13). Nonetheless, TAVR tends to outperform SAVR in terms of infective endocarditis (14). Retrospective observational studies based on medical registries or hospital data have reported comparable clinical outcomes for the two procedures in terms of mortality and major adverse cardiac and cerebrovascular events (15–19); however, the availability of short or mid-term follow-up data has been limited.

Randomized controlled trials and observational studies have demonstrated the efficacy of TAVR within a selected cohort of patients and hospital centers; however, there has been limited research on the long-term dissemination and utilization of TAVR vs. SAVR in routine clinical practice. Previous studies pertaining to the cost of AS care have yielded inconsistent results. In general, the initial costs of TAVR are higher than those of SAVR; however, the utilization of post-procedure resources tends to be lower, with follow-up costs proportional to risk at the patient level. Essentially, researchers have yet to elucidate the actual costs associated with TAVR and SAVR over various post-procedure periods.

This study compared TAVR and SAVR in terms of effectiveness, medical utilization, and medical costs during the procedure and in various post-procedure phases.

## Materials and methods

### Study design and data source

This non-interventional, retrospective cohort study compared TAVR and SAVR in terms of clinical outcomes and medical utilization in a real-world setting. The single-payer mandatory National Health Insurance (NHI) program currently covers more than 99% of the 23 million residents of Taiwan. The NHI claims database comprises all longitudinal medical claims data from insured individuals, including ambulatory visits, hospital admissions, procedures, medication, rehabilitation, and home care since 1995. This study linked national NHI claims data and Cause of Death data from the Health and Welfare Data Science Center for the period 2015–2019.

This study was approved by the Institutional Review Board of National Yang Ming Chiao Tung University (IRB no. YM110048E).

### Study cohort

**Figure 1:**
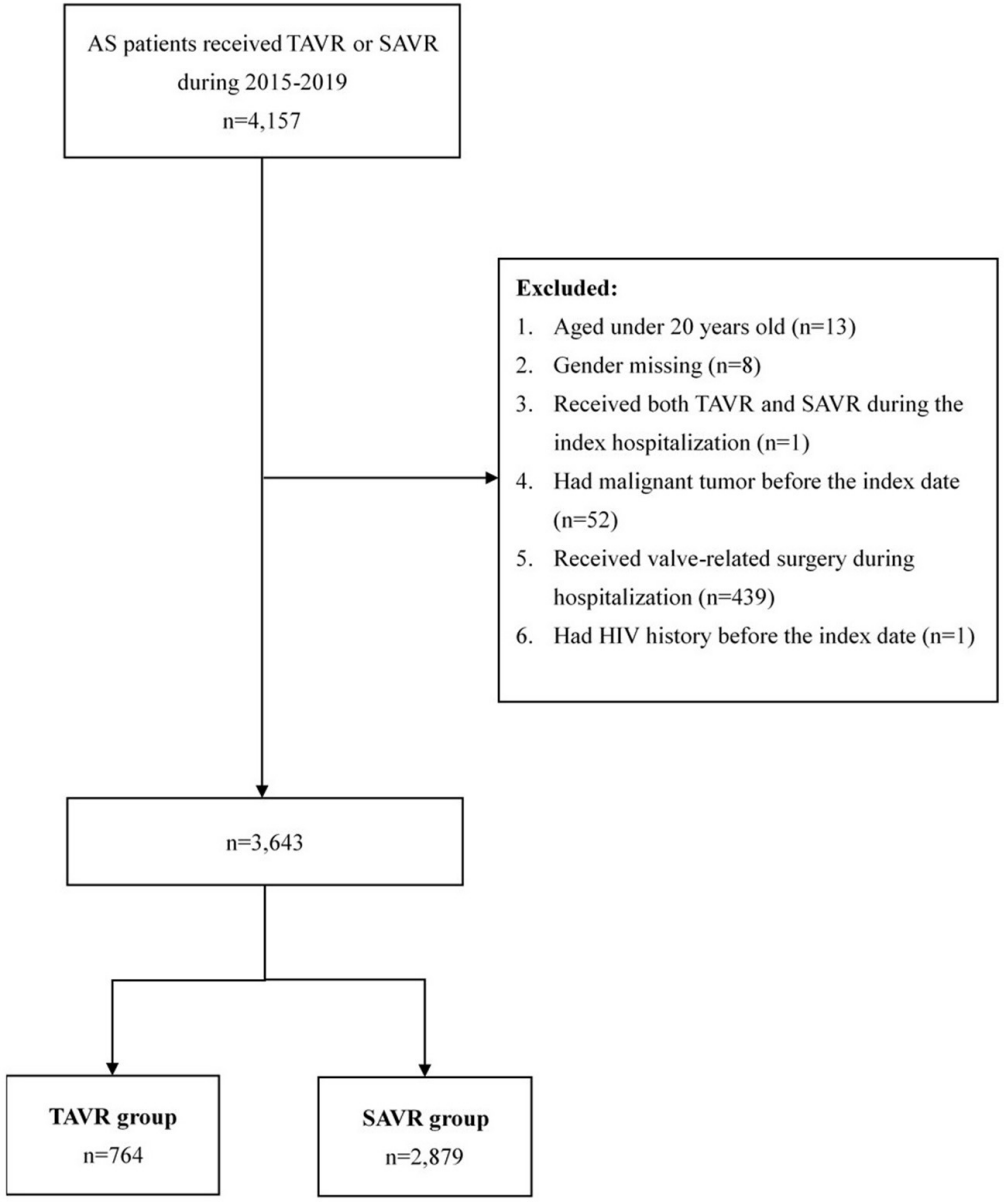
Study Flow. Patient selection in the current study.

Patients were diagnosed with AS based on the International Classification of Diseases, Ninth Revision, Clinical Modification, and Tenth Revision, Clinical Modification (ICD-9-CM and ICD-10-CM) (Supplementary Table 1). We recruited a total of 4,157 patients with AS who had undergone TAVR (*n* = 505) or SAVR (*n* = 4,157) during the study period (Figure 1). We excluded patients <20 years (*n* = 13), those who were missing gender data (*n* = 8), those who received both TAVR and SAVR during index hospitalization (*n* = 1), those who presented a malignant tumor before treatment (*n* = 439), those who received valve-related surgery during the index hospitalization, and those with a history of HIV (*n* = 1). A final study cohort of 3,643 patients was included in our analysis.

The first TAVR procedure in Taiwan was performed as a clinical trial in 2010 and the first TAVR device was approved by the Taiwan FDA in 2012. Early valve technologies included CoreValve (Medtronic Inc., Minneapolis, MN), Lotus (Boston Scientific, Natick, MA), and Sapien XT (Edwards Lifesciences, Irvine, CA), which were launched, respectively, in 2012, 2015, and 2016. New-generation TAVR devices, including Evolut R (Medtronic Inc., Minneapolis, MN), Sapien 3 (Edwards Lifesciences, Irvine, CA) and Portico (Abbott Vascular Inc., Santa Clara, CA), all of which were introduced in 2017 (20). Most of the TAVR operations during the study period (2015–2019) involved new-generation devices.

### Variable definitions

Primary outcomes included all-cause mortality (ACM), hospitalization for heart failure (HHF), and a composite major adverse cardiovascular event (MACE), including myocardial infarction (MI), stroke, and cardiovascular (CV) death. We also evaluated individual outcomes of MI, stroke, and CV death. The follow-up time was defined as the interval between TAVR or SAVR (index hospitalization) and the date of death, as recorded in the Cause of Death data or the date of observed outcomes.

Secondary outcomes in this study included the medical utilization and costs associated with TAVR or SAVR in the index admission patient receiving the procedure and in various periods after the procedure. The length of stay and hospitalization cost at index admission were estimated for cost analysis. Medical utilization related to AS, including the number of outpatient visits, length of stay, cost of outpatient visit, admission cost, and total medical cost, were aggregated for various post-procedure periods. We also evaluated cumulative medical costs, number of outpatient visits, and length of stay after the procedure.

The primary independent variable was the treatment strategy (TAVR or SAVR). Covariates included patient age, gender, treatment year, marital status, education level, Elixhauser comorbidity index, hospital frailty risk score (21), dialysis, hypertension, received percutaneous coronary dilation during the 365 days prior to index hospitalization, concomitant medications (lipid-lowering therapies, antiplatelet, anticoagulants, non-steroidal anti-inflammatory drug (NSAID), angiotensin-converting enzyme inhibitor, angiotensin receptor blockers, antihypertensive medications, and anti-diabetes medications), and ownership and accreditation level of hospital at which the patient received treatment. The definitions of comorbidities and concomitant medications are listed in the Supplementary Tables 1, 2.

### Statistical analysis

Categorical and ordinal variables are presented as frequency and continuous variables are presented as mean and standard division (SD). Propensity scores based on the above variables were used to account for confounding by intervention. Overlap weighting was also used to minimize the influence of extreme propensity scores in individuals (22–24). Standardized differences (StD) between tcovariates of these two groups were compared before and after propensity score (PS) overlap weighting (25).

The cumulative incidence of adverse health outcomes was estimated using Kaplan-Meier survival curves based on propensity score overlap weighting. The Cox proportional hazard model with a robust estimator was used to evaluate the association of TAVR vs. SAVR treatment with ACM using propensity score overlap weighting. In evaluating health outcomes other than ACM (i.e., HHF, MI, stroke, and CV death), the Fine and Gray proportional sub-distribution hazard model with PS overlap weighting was used to account for competing risk of death. Adjusted hazard ratios for health outcomes are presented. Prespecified subgroup analysis was conducted according to age group (<70 years and ≧70 years), gender, comorbidities, prevalent dialysis, hypertension, and hospital frailty risk score. The proportional hazard assumptions were assessed using a graphic plot of ln{−*ln*[*S*(*t*)]} curves of the two treatment groups, wherein the appearance of reasonably parallel lines indicated no violation. In sensitivity analysis, we applied landmark estimates obtained using the Kaplan-Meier method with an 18-month grace period after the procedure to avoid immortal-time bias and reverse causation.

Generalized estimation equation (GEE) models were used to estimate the effects of the treatment strategy on the number of outpatient visits, length of stay, and costs. All costs are presented in US dollars ($) based on an exchange rate from TWD of 1:28. All *p*-values were two-sided and *P* < 0.05 was considered statistically significant. The syntax “PROC PHREG” was used to analyze health outcomes with time to event, while “PROC GENMOD” was used to analyze medical utilization and costs. All analyses were performed using SAS version 9.4 (SAS, Gray, North Carolina).

## Results

### Participant characteristics

A total of 3,643 patients were involved in the analytic dataset, including 764 who underwent TAVR and 2,879 who underwent SAVR. The mean age was 77.98 years (SD = 5.86), a small majority (53.94%) were women, and 22.07% of the patients had previously received percutaneous coronary dilatation. The mean Elixhauser comorbidity index was 1.46 (SD = 0.96). As for hospital level, 38.79% of the patients received treatment in a public hospital and 66.42% received treatment in a medical center. Table 1 presents the demographics of patients at baseline before and after propensity score overlap weighting.

**Table 1:** Baseline characteristics of patients before and after propensity score overlap weighting.

	**Unweighted**			**After propensity score weighting**		
	**TAVR**	**SAVR**	**StD**	**TAVR**	**SAVR**	**StD**
	**(*n* = 764)**	**(*n* = 2,879)**				
**Year of treatment, %**						
2015	15.84	25.04	0.143	18.52	18.52	0.000
2016	20.55	18.27		22.03	22.03	
2017	21.47	17.75		20.20	20.20	
2018	21.73	19.45		20.41	20.41	
2019	20.42	19.49		18.84	18.84	
Age, mean (SD)	81.32(7.76)	66.97(11.76)	1.440	77.98(5.86)	77.98(2.55)	0.000
**Gender, %**						
Male	46.07	56.58	−0.211	46.06	46.06	0.000
Female	53.93	43.42		53.94	53.94	
**Marital status, %**						
Unmarried	1.18	5.52	0.335	1.63	1.63	0.000
Married	57.85	67.14		61.12	61.12	
Divorced, widowed, or others	40.97	27.34		37.25	37.25	
**Educational level, %**						
Bachelor's, master's, and doctoral degree	70.94	68.08	−0.035	76.19	76.19	0.000
High school graduate	17.15	22.96		15.41	15.41	
Others	11.91	8.96		8.40	8.40	
**Comorbidity in 365 days before the index hospitalization, %**						
Elixhauser comorbidity index, mean (SD)	1.58(1.41)	1.02(1.27)	0.420	1.46(0.96)	1.46(0.53)	0.000
Hospital frailty risk score, mean (SD)	0.79(1.50)	1.26(1.90)	0.279	1.15(1.24)	1.18(0.66)	−0.032
Median (Q1–Q3)	0(0–1.4)	0(0–2.05)		0(0–1.8)	0(0–1.8)	
Dialysis	22.12	18.51	0.090	23.94	23.94	0.000
Hypertension	27.88	19.31	0.203	27.38	27.38	0.000
Received PCI/CABG	31.54	10.80	0.525	22.07	22.07	0.000
Concomitant medication in 365d before the index hospitalization, %						
Statins	54.06	46.82	0.145	54.26	54.26	0.000
Other lipid-lowering drugs, excluding statins	4.71	5.18	−0.021	5.85	5.85	0.000
Antiplatelet	76.05	62.52	0.296	73.67	73.67	0.000
Anticoagulant	18.59	9.62	0.260	15.43	15.43	0.000
NSAID	66.88	71.38	−0.097	68.88	68.88	0.000
ACEI	15.45	15.46	0.000	15.43	15.43	0.000
ARB	62.70	53.00	0.197	61.16	61.16	0.000
Beta blocker	65.97	59.92	0.126	65.16	65.16	0.000
Calcium channel blocker	64.92	51.34	0.278	62.29	62.29	0.000
Thiazide	14.4	6.88	0.246	11.58	11.58	0.000
Loop diuretic	61.26	43.49	0.362	55.42	55.42	0.000
Metformin	15.58	14.90	0.019	15.98	15.98	0.000
Oral hypoglycemic agent	31.15	24.66	0.145	30.66	30.66	0.000
Insulin	3.53	3.02	0.029	3.37	3.37	0.000
**Hospital ownership, %**						
Public hospital	42.02	38.35	−0.046	38.78	38.78	0.000
Private hospital	8.38	11.11		9.15	9.15	
Non-profit hospital	49.61	50.54		52.07	52.07	
**Hospital accreditation level, %**						
Medical center	67.67	69.95	0.045	66.42	66.42	0.000
Regional hospital	32.33	29.45		33.58	33.58	
Local hospital	0	0.59		0.00	0.00	

TAVR, transcatheter aortic valve replacement; SAVR, surgical aortic valve replacement; StD, standardized difference; NSAID, non-steroidal anti-inflammatory drug; ACEI, angiotensin-converting enzyme inhibitor; ARB, angiotensin receptor blocker.

### Clinical outcomes

**Figure 2:**
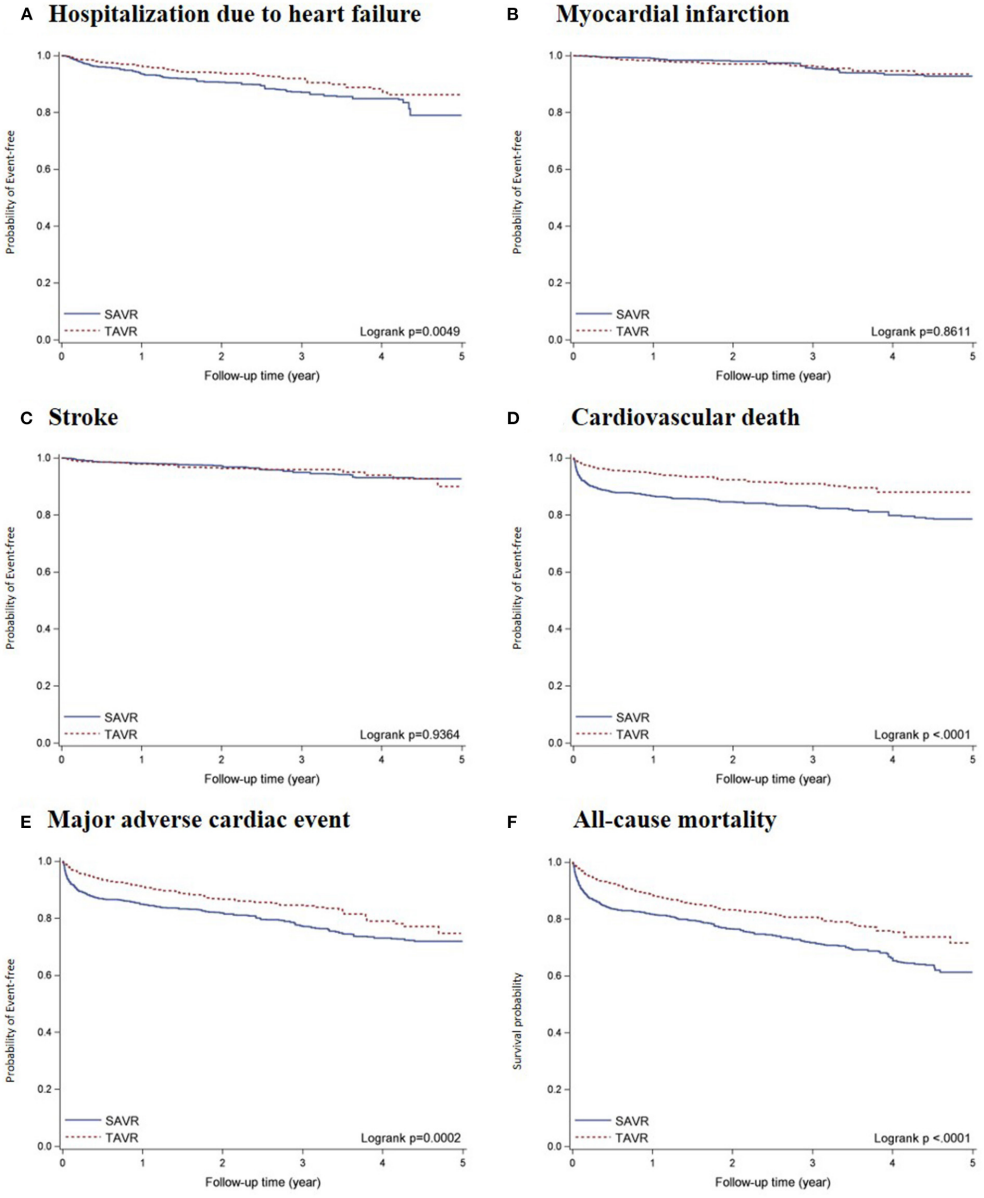
Kaplan-Meier survival curves of all-cause mortality and incident CV-related outcomes comparing TAVR vs. SAVR after propensity score overlap weighting: **(A)** HHF; **(B)** MI; **(C)** Stroke; **(D)** CV death; **(E)** MACE; and **(F)** ACM. Results of Kaplan-Meier survival analysis of primary and secondary outcomes after propensity score overlap weighting.

Within a median follow-up of 2.02 years (Q1–Q3: 0.81–3.49; mean ± SD: 2.20 ± 1.51), a total of 162 deaths occurred, including 88 deaths due to CV incidents. In-hospital mortality was higher among patients who underwent SAVR than among those who underwent TAVR (10.92% vs. 3.83%), and 30-day mortality after discharge was also higher among patients who underwent SAVR (8.08% vs. 2.26%) (Supplementary Table 3). Prior to adjustment, the TAVR group had a lower percentage of patients free from hospitalization due to heart failure (HHF) (*P* = 0.0049), major adverse cardiovascular event (MACE) (*P* = 0.0002), cardiovascular death (*P* < 0.0001), and all-cause mortality (*P* < 0.0001; Figure 2). The landmark estimates of all-cause mortality at 18 months were consistent (Supplementary Figure 1).

The total number of deaths per 1,000 person-years were 82.40 and 130.51 in the TAVR and SAVR groups, respectively. The number of cardiovascular deaths per 1,000 person-years were 36.54 and 79.27 in the TAVR and SAVR groups, respectively. The weighted rates of MACE per 1000 person-years were 65.91 and 101.65 in the TAVR and SAVR groups, respectively. After propensity score overlap weighting, TAVR was significantly associated with a lower risk of all-cause mortality (HR, 0.61 [95% CI, 0.44–0.84]), major adverse cardiovascular event (HR, 0.66 [95%, 0.46–0.96], and cardiovascular death (HR, 0.47 [95% CI, 0.30–0.74]) (Table 2). We observed no significant differences between the TAVR and SAVR groups in terms of the risk of hospitalization due to heart failure, myocardial infarction, or stroke.

**Table 2:** Comparison of TAVR and SAVR in terms of health outcomes in 3,643 AS patients after propensity score overlap weighting.

	**No. of events (%)**		**Follow-up period (PYs)**		**Weighted rate/** **1,000 PYs**	**adj-HR (95% CI)**	***P***
			**mean (SD)**	**median (Q1–Q3)**			
**HHF**
SAVR	34	9.06	1.84 (0.52)	1.66 (0.46–2.98)	49.26	1.00 [Reference]	
TAVR	25	6.66	2.04 (1.01)	1.79 (0.73–3.16)	32.65	0.78 (0.46–1.33)	0.3654
**MI**
SAVR	10	2.62	1.94 (0.53)	1.77 (0.59–3.04)	13.53	1.00 [Reference]	
TAVR	11	2.96	2.08 (1.01)	1.95 (0.78–3.24)	14.24	1.33 (0.54–3.25)	0.5340
**Stroke**
SAVR	12	3.23	1.93 (0.53)	1.77 (0.56–3.05)	16.75	1.00 [Reference]	
TAVR	13	3.53	2.07 (1.01)	1.90 (0.78–3.24)	17.06	1.28 (0.56–2.93)	0.5649
**CV death**
SAVR	59	15.62	1.97 (0.53)	1.80 (0.60–3.10)	79.27	1.00 [Reference]	
TAVR	29	7.71	2.11 (1.01)	1.99 (0.82–3.28)	36.54	0.47 (0.30–0.74)	0.0011
**MACE**
SAVR	73	19.42	1.91 (0.52)	1.76 (0.56–3.01)	101.65	1.00 [Reference]	
TAVR	51	13.51	2.05 (1.01)	1.80 (0.75–3.18)	65.91	0.66 (0.46–0.96)	0.0274
**ACM**
SAVR	97	25.71	1.97 (0.53)	1.80 (0.60–3.10)	130.51	1.00 [Reference]	
TAVR	65	17.39	2.11 (1.01)	1.99 (0.82–3.28)	82.40	0.61 (0.44–0.84)	0.0022

TAVR, transcatheter aortic valve replacement; SAVR, surgical aortic valve replacement; HHF, hospitalization due to heart failure; MI, myocardial infarction; CV, cardiovascular; MACE, major adverse cardiac event; ACM, all-cause mortality.

**Figure 3:**
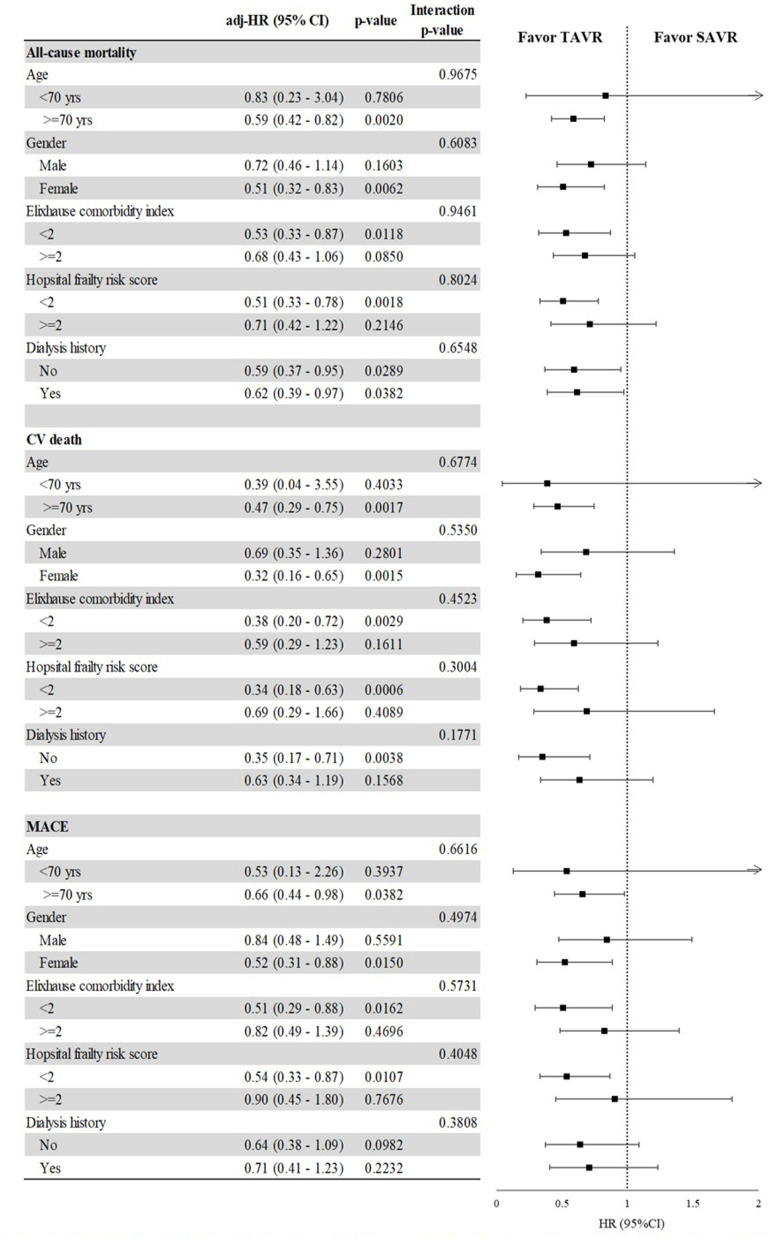
The impact of the interaction between selected categories and TAVR vs. SAVR on the risk of all-cause mortality, CV death, and MACE, after propensity score overlap weighting. Comparison of TAVR and SAVR in terms of MACE, CV death, and ACM among selected baseline characteristics after propensity score overlap weighting. Subgroup analysis comparing TAVR and SAVR as a function of age group, gender, comorbidity score, hospital frailty risk score, and history of dialysis. Outcomes included in the subgroup analysis were all-cause mortality, cardiovascular death, and major adverse cardiovascular event.

In subgroup analysis (Figure 3), the risks of all-cause mortality, CV death, and MACE were significantly lower in the TAVR group than in the SAVR group for patients aged >70 years, females, and those with a low Elixhauser comorbidity index, regardless of the hospital frailty risk score. TAVR was also associated with a lower risk of all-cause mortality among patients with a history of dialysis (HR, 0.59 [95% CI, 0.37–0.95]) as well as among those without a history of dialysis (HR, 0.62 [95% CI, 0.39–0.97]).

### Medical utilization and costs

The medical utilization and costs at admission and in post-procedure periods are reported in Table 3. The mean length of stay during the index hospitalization was shorter in the TAVR group than in the SAVR group (19.20 ± 14.37 days vs. 29.50 ± 9.61 days, *P* < 0.0001). Hospitalization costs were significantly lower in the TAVR group ($14,016.81 ± 8,460.95) than in the SAVR group ($22,752.06 ± 5,835.91) (*P* < 0.0001). The mean aggregated total medical costs, including all ambulatory visits and all admissions in the first year after treatment, were $1,271.89 (SD = 4,048.36) in the TAVR group and $887.20 (SD = 978.51) in the SAVR group (*P* = 0.0138). However, note that we did not observe a significant difference between the TAVR and SAVR groups in terms of total medical costs in the second (*P* = 0.1256) or third year (*P* = 0.5997) after treatment. Cost associated with outpatient visits in the second year was higher in the TAVR group than in the SAVR group ($393.60 ± 812.47 vs. $299.68 ± 218.77; *P* = 0.0425).

**Table 3:** Medical utilization during surgery and in different post-procedure periods after propensity score overlap weighting.

	**TAVR**	**SAVR**	**difference (TAVR-SAVR)**	***P***	**adj-difference (TAVR-SAVR)**	***P***
	**Mean (SD)**	**Mean (SD)**	**Estimate (SE)**		**Estimate (SE)**	
**Medical utilization of index hospitalization**						
Length of stay (day)	19.20 (14.37)	29.50 (9.61)	−10.30 (0.89)	<.0001	−10.24 (0.73)	<.0001
Median (Q1–Q3)	13 (8–12)	21 (14–35)
Hospitalization cost (US$)	14,016.81 (8,460.95)	22,752.06 (5,835.91)	−8,735.25 (530.07)	<.0001	−8,711.25 (423.46)	<.0001
**Medical utilization in 1st year after index hospitalization**						
No. of outpatient visits	7.88 (5.71)	7.55 (2.82)	0.33 (0.28)	0.2262	0.33 (0.27)	0.2164
Length of stay (day)	1.01 (5.98)	1.48 (3.61)	−0.47 (0.32)	0.1428	−0.55 (0.31)	0.0755
Outpatient visit cost (US$)	747.41 (678.07)	495.61 (235.24)	251.80 (28.51)	<.0001	252.34 (27.76)	<.0001
Hospitalization cost (US$)	524.48 (3,977.36)	391.59 (948.42)	132.89 (153.05)	0.3853	87.12 (150.24)	0.5620
Total medical cost (US$)	1,271.89 (4,048.36)	887.20 (978.51)	384.69 (156.16)	0.0138	339.47 (153.11)	0.0266
**Medical utilization in 2nd year after index hospitalization**						
No. of outpatient visit	8.18 (5.81)	8.09 (2.79)	0.09 (0.36)	0.8012	0.13 (0.28)	0.6454
Length of stay (day)	0.12 (1.00)	0.30 (1.08)	−0.18 (0.08)	0.0270	−0.18 (0.08)	0.0322
Outpatient visit cost (US$)	393.60 (812.47)	299.68 (218.77)	93.92 (46.22)	0.0425	88.94 (31.83)	0.0052
Hospitalization cost (US$)	43.20 (349.78)	76.25 (280.50)	−33.05 (24.95)	0.1854	−29.64 (23.22)	0.2017
Total medical cost (US$)	436.80 (877.04)	375.93 (352.32)	60.87 (52.07)	0.2427	59.29 (38.71)	0.1256
**Medical utilization in 3rd year after index hospitalization**						
No. of outpatient visit	2.48 (3.41)	2.50 (2.00)	−0.02 (0.22)	0.9361	−0.07 (0.17)	0.6653
Length of stay (day)	0.08 (1.00)	0.34 (2.27)	−0.26 (0.14)	0.0681	−0.26 (0.17)	0.1127
Outpatient visit cost (US$)	265.10 (787.76)	188.39 (191.54)	76.71 (45.31)	0.0909	70.08 (30.52)	0.0217
Hospitalization cost (US$)	24.30 (293.34)	70.89 (346.97)	−46.59 (25.99)	0.0732	−48.74 (26.98)	0.0708
Total medical cost (US$)	289.40 (846.25)	259.28 (399.06)	30.12 (52.59)	0.5669	21.34 (40.66)	0.5997
**Cumulative medical utilization after index hospitalization**						
Total medical cost (US$)	2,078.12 (4,480.30)	1,558.62 (1,368.77)	489.50 (247.20)	0.0480	420.49 (176.48)	0.0172
Total outpatient visits	14.96 (12.96)	14.85 (7.27)	0.11 (0.79)	0.8906	−0.09 (0.62)	0.8857
Total length of stay	1.26 (6.21)	2.22 (6.41)	−0.97 (0.48)	0.0438	−1.06 (0.47)	0.0245

In the multivariate analysis (Table 3), the length of stay (adjusted difference: $-10.24 ± 0.73, *P* < 0.0001) and the corresponding hospitalization costs (adjusted difference: $-8,711.25 ± 423.46, *P* < 0.0001) of index admission were lower for patients who underwent TAVR than for those who underwent SAVR. The cost of outpatient visits in the first, second, and third years after treatment was significantly higher for patients who underwent TAVR than for those who underwent SAVR. Furthermore, total medical costs in the first year after treatment were higher for the TAVR group than for the SAVR group (adjusted difference: $339.47 ± 153.11, *P* = 0.0266), due to higher costs for outpatient visits in the TAVR group. No significant differences were observed between the TAVR and SAVR groups in terms of total medical cost in the second and third years after the procedure.

During the 5-year follow-up period, the cumulative total medical costs associated with TAVR ($2,078.12 ± 4,480.30) were slightly higher than those of SAVR ($1,558.62 ± 1,368.77; *P* = 0.0480). After adjustment for other covariates, cumulative medical costs were significantly higher in the TAVR group than in the SAVR group (adjusted difference: $420.49 ± 176.48, *P* = 0.0172), whereas the cumulative length of stay was shorter in the TAVR group (adjusted difference:−1.06 ± 0.47, *P* = 0.0245).

## Discussion

This study compared TAVR and SAVR for the treatment of AS based on a large nationwide claim database. To date, this is the most extensive and comprehensive report on patient demographics, clinical outcomes, and medical utilization during and after treatment. The principal findings of this study can be summarized as follows: (1) TAVR is superior to SAVR in terms of overall survival, CV-related survival, and MACE; and (2) TAVR shortens the length of stay which reduced hospitalization costs during the procedure but had slightly higher cumulative medical cost after the procedure.

Observational data suggest that TAVR is superior to SAVR in terms of mortality. Based on the National Readmission Database, Lemor et al. reported that TAVR was superior to SAVR in terms of in-hospital mortality rate, 30-day mortality, and 30-day readmission rate (17). Based on a nationwide registry in Finland, Virtanen et al. reported that TAVR and SAVR were similar in terms of 30-day mortality and 3-year survival (18). In our 5-year follow-up of the current study, the risk of patients experiencing MACE was 34% lower in the TAVR group compared to the SAVR group, the risk of CV death was 53% lower, and the risk of ACM was 39% lower. Our landmark analysis on outcomes for the period 18 months to 5 years after the procedure revealed that TAVR was associated with a lower likelihood of all-cause death. The low incidence of mortality in the TAVR group during this time period may be attributed to a lower incidence of bleeding, transfusion, and post-operative complications (3, 14, 18, 26).

Advanced kidney disease was identified as a risk factor for patients in both groups and a significant predictor of mortality for patients in the TAVR group (27). Previous studies reported that the short-term survival benefits of TAVR therapy are also applicable to patients with chronic kidney disease or end-stage renal disease (28–30). We also found that within a 5-year follow-up period, the risk of all-cause mortality in the TAVR group was 38% lower among patients with a history of dialysis and 41% lower among those without a history of dialysis. As in previous studies, we determined that the benefits of TAVR could extend to patients with or without advanced kidney disease who did not undergo surgery. In the current study, TAVR was associated with a lower risk of all-cause mortality, CV death, and major adverse cardiovascular events among patients with a relatively low hospital frailty score. This result was consistent with previous studies showing that frail patients inevitably face an elevated risk of mortality after receiving TAVR (31–33). All-cause mortality at 1 year after TAVR in this study (11.39%) was lower than that of the high-risk patient in the PARTNER I trial (24.2%) (3). Although the inclusion criteria for the PARTNER I trial and Taiwan's NHI reimbursement criteria for TAVR were similar, the patients in our study were younger (77.98 vs. 83.6 years) and had less comorbidity than those included in the PARTNER I trial, which may be the possible reason for the lower mortality at 1 year in the current study. However, the all-cause mortality of the TAVR group at 1 year and 2 years in our study was similar to the PARTNER II trial (11.39 vs. 12.3% at 1 year; 16.74 vs. 16.7% at 2 years) (6, 8). In Taiwan, the NHI reimbursed TAVR to high-risk patients; however, partial intermediate-risk patients could receive TAVR if they are over 80 years.

In the current study, we found that the length of stay at the index admission patient received treatment was significantly shorter in the TAVR group than in the SAVR group, which was consistent with previous studies (34–36). We also found that the hospitalization cost was 1.6 times higher for SAVR patients than for TAVR patients, and the difference remained significant after adjusting for baseline characteristics. Based on electronic health records in Germany, Kaier et al. (37) reported that the cost of hospitalization for TAVR (mean ± SD, 33,936 ± 6,601) was higher than the costs for SAVR (mean±SD, 19,055 ± 11,976). In 2012, Medicare payments for 4,083 TAVR patients [median, $50,200; interquartile range (IQR), $39,800–64,300)] was slightly higher than that for SAVR patients (median, $45,500; IQR, $34,500–63,300; *P* < 0.01) in a propensity-matched population. These findings can be attributed to differences in patient populations, analytic perspectives, and other factors. First, the cost of TAVR was higher than that of SAVR; however, payments for the implanted valve prosthesis was partially covered by the NHI in Taiwan. In addition, non-procedure costs were lower due largely to significantly shorter in-hospital stays (35).

Few studies have compared costs over the long term. Based on the Nationwide Readmissions Database in the US, Glodsweig et al. estimated the inpatient costs for 6 months (36). They found that the total admission costs associated with TAVR ($10,996) were slightly higher than those of SAVR ($7,285). Analysis related to the cost-effectiveness of the PARTNER II trial revealed that 1-year follow-up costs of TAVR were significantly lower than those for SAVR (risk-adjusted difference: $11,377; *P* < 0.001) (35). In the current study, the outpatient visits cost and total medical costs at first year after treatment were higher for TAVR than for SAVR; however, we did not observe a significant difference in total medical costs in the second or third year after the procedure. This balancing of costs can possibly be attributed to a shorter length of stay and a lower hospitalization cost in the TAVR group. The higher cumulative medical costs in the TAVR cohort during the post-procedure period can be attributed to higher medical costs in the first year and higher costs for outpatient visits in the second and third years after the procedure. In current study, more than 90% of the total medical cost of TAVR group in the second and third years after treatment was contributed by the cost of the outpatient visit. Patients generally require frequent checkups and imaging tests to verify that the device is operating properly after TAVR, which may be the potential reason for the higher outpatient visit cost of TAVR than SAVR.

Our findings contribute to an understanding of short- and long-term clinical outcomes of these procedures as well as cumulative medical utilization and costs. Claims data are widely used to define a cohort of patients, and procedural and diagnostic codes are used to accurately determine the corresponding health outcomes (38). One of the benefits of using data from routine clinical practice is the availability of large amounts of patient-level information by which to capture relevant characteristics. This is particularly important for patients with complex medical conditions, many of whom are excluded from trials due to comorbidities (e.g., end-stage renal disease, previous peripheral intervention, or dementia) and concomitant medications (e.g., anticoagulant regimens) (6), which puts them at increased risk of cardiovascular events and death. We gained a number of insights through our use of propensity score overlap weighting to minimize variance in the correlations between TAVR and SAVR. Propensity score matching has been widely used in previous observational studies to adjust for differences in measured characteristics; however, the effectiveness of these methods is limited in situations where initial differences between groups are large and do not achieve good balance or have worse precision (39). In the current study, we also evaluated follow-up costs based on NHI claims data and the corresponding payments. There is a high probability that this approach is able to capture follow-up costs that might otherwise be overlooked (particularly costs associated with rehabilitation, home care, and outpatient services). Accordingly, we were able to determine that long-term follow-up costs for TAVR were comparable to those for SAVR, which has not previously been reported (40, 41).

The current study has several limitations. First, observational studies are unable to provide conclusions as strong as those obtained using randomized controlled trials, due to residual confounding factors and the fact that treatments are not randomly assigned. In the current study, we used propensity score overlap weighting to minimize selection bias between groups; however, the risk of confounding variables cannot be excluded. We also employed an administrative follow-up scheme to minimize loss to follow-up. Second, our use of claim data also introduced inevitable coding errors. Nonetheless, we sought to reduce misclassification bias by linking NHI claims data with Cause of Death data based on scrambled identification to identify instances of death. We also used procedural billing codes to facilitate endpoint identification (38). Third, the dataset used in the current study lacked relevant clinical information related to STS, EureSCORE, and echocardiographic findings, all of which could have an impact on the severity of the disease. It is important to note that TAVR is reimbursed by the NHI; however, AS patients who receive TAVR must meet the following requirements: (a) New York Heart Association Function Class II-IV; (b) Aortic area (AVA) of 0.8 cm^2^ or an AVA index of ≦0.6 cm^2^/m^2^, and either a mean gradient ≧40 mm Hg or peak aortic jet velocity > 4 m/s; (c) Excessive risk for open-heart surgery, as designated by at least two cardiac surgery doctors; (d) STS Score >10% or Logistic EuroSCORE I >20%, or 80 years and older, or previous history of cardiac surgery (CABG or valve-related surgery), serious porcelain aorta, liver cirrhosis (Child A or B), or lung insufficiency (FEV < 1 ml). Patients with AS who received SAVR in Taiwan were mostly intermediate to low risk. Therefore, we used the Elixhauser comorbidity index, hospital frailty risk scores, comorbidities (including dialysis, previous percutaneous coronary dilation, or arterial endarterectomy), and concomitant medication (including insulin) as an indirect adjustment for severity. The Elixhauser comorbidity index includes 31 comorbidities, including congestive heart failure, cardiac arrhythmias, valvular disease, pulmonary circulation disorders, chronic pulmonary disease, complicated diabetes, renal failure, and coagulopathy (42, 43). The hospital frailty score is a significant predictor of all-cause mortality and rehospitalization among patients receiving TAVR (33, 44). Through these means, we may adjust the severity indirectly and our results reported survival benefit of TAVR with comparable post-procedure costs. Finally, reimbursements pertaining to TAVR and SAVR differ among different healthcare systems, so that our results are not necessarily generalizable to other countries.

To summarize, our 5-year data comparing TAVR or SAVR for AS in terms of outcomes and medical costs revealed that TAVR is superior to SAVR in terms of survival benefits and comparable follow-up costs. Our findings suggest that TAVR may be a better treatment strategy for AS based on clinical and economic considerations.

## Data availability statement

The data analyzed in this study is subject to the following licenses/restrictions: Data used in this study was obtained from the Health and Welfare Data Science Center, Department of Statistics, Ministry of Health and Welfare. Researchers and doctors in Taiwan could apply to the data and analyze it on-site. Requests to access these datasets should be directed to https://dep.mohw.gov.tw/dos/cp-5119-59201-113.html.

## Ethics statement

The studies involving human participants were reviewed and approved by Institutional Review Board of National Yang Ming Chiao Tung University (IRB no: YM110048E). Written informed consent for participation was not required for this study in accordance with the national legislation and the institutional requirements.

## Author contributions

ET, Y-TL, YK, T-PT, K-CLe, M-CH, JW, K-CLi, and W-HY: conception and design. ET, Y-TL, YK, K-CLi, and W-HY: analysis and interpretation of data. ET, Y-TL, YK, and W-HY: drafting the manuscript. ET, K-CLi, and W-HY: final approval of the manuscript. All authors contributed to the article and approved the submitted version.

## Funding

This study was supported by the National Yang Ming Chiao University and Cheng Hsin General Hospital (Grant No. CY11001).

## Conflict of interest

The authors declare that the research was conducted in the absence of any commercial or financial relationships that could be construed as a potential conflict of interest.

## Publisher's note

All claims expressed in this article are solely those of the authors and do not necessarily represent those of their affiliated organizations, or those of the publisher, the editors and the reviewers. Any product that may be evaluated in this article, or claim that may be made by its manufacturer, is not guaranteed or endorsed by the publisher.
