# Supplementary material for: Clinical outcomes and cumulative healthcare costs of TAVR vs. SAVR in Asia
Source: Front Cardiovasc Med. 2022 Sep 21;9:973889. doi: 10.3389/fcvm.2022.973889 (PMC9532629; doi:10.3389/fcvm.2022.973889)
Supplement: Supplementary file 1 [file Table_1.PDF]

Supplementary materials

Supplemental Table 1. Description of ICD codes from claims
Supplemental Table 2. Description of ATC order codes from claims
Supplemental Table 3. In-hospital and 30-day mortality between TAVR and SAVR after propensity score overlap weighting
Supplementary Figure 1. Landmark analysis: primary and secondary outcomes from the first year after surgery, including HHF (A), CV death (B), MACE (C), and all-cause mortality (D).

Supplemental Table 1. Description of ICD codes from claims

Disease/Outcome	ICD-9 Code	ICD-10 Code	Description
Aortic stenosis	395.0	I06.0	Rheumatic aortic stenosis
	395.2	I06.2	Rheumatic aortic stenosis with insufficiency
	396.0		Mitral valve stenosis and aortic valve stenosis
	396.2		Mitral valve insufficiency and aortic valve stenosis
		I08.0	Rheumatic disorders of both mitral and aortic valves
	424.1		Aortic valve disorders
		I35.0	Nonrheumatic aortic (valve) stenosis
		I35.2	Nonrheumatic aortic (valve) stenosis with insufficiency
	746.3	Q23.0	Congenital stenosis of aortic valve
Hypertension	401	I10	Essential (primary) hypertension
	402	I11	Hypertensive heart disease
	403	I12	Hypertensive chronic kidney disease with chronic kidney disease or renal disease
	404	I13	Hypertensive heart and chronic kidney disease with heart failure or renal disease
	405	I15<br>N26.2	Secondary hypertension
Heart failure	398.91	I09.81	Rheumatic heart failure (congestive)
	402.01		Malignant hypertensive heart disease with congestive heart failure
	402.11		Benign hypertensive heart disease with congestive heart failure
	402.91		Unspecified hypertensive heart disease with congestive heart failure
		I11.0	Hypertensive heart disease with heart failure

Disease/Outcome	ICD-9 Code	ICD-10 Code	Description
	404.01		Malignant hypertensive heart and renal disease with congestive heart failure
	404.03		Malignant hypertensive heart and renal disease with congestive heart failure and renal failure
	404.11		Benign hypertensive heart and renal disease with congestive heart failure
	404.13		Benign hypertensive heart and renal disease with congestive heart failure and renal failure
	404.91		Unspecified hypertensive heart and renal disease with congestive heart failure
	404.93		Unspecified hypertensive heart and renal disease with congestive heart failure and renal failure
		I13.0	Hypertensive heart and chronic kidney disease with heart failure and stage 1 through stage 4 chronic kidney disease, or unspecified chronic kidney disease
		I13.2	Hypertensive heart and chronic kidney disease with heart failure and with stage 5 chronic kidney disease, or end stage renal disease
	428.x	I50.x	Congestive heart failure
Myocardial infarction	410.x1	I21-I22	Acute myocardial infarction
Stroke	430	I60	Subarachnoid hemorrhage
	431	I61	Intracerebral hemorrhage
	433	I63	Occlusion and stenosis of artery
	434	I63	Cerebral thrombosis, cerebral embolism or cerebral artery occlusion
	435	G45	Artery syndrome or transient cerebral ischemia
	436	I67.89	Other cerebrovascular disease
Cardiovascular		I00	Rheumatic fever without heart

**Disease/Outcome**	**ICD-9 Code**	**ICD-10 Code**	**Description**
causes of death			involvement
		I01	Acute rheumatic heart disease
		I02	Rheumatic chorea
		I05-I09	Rheumatic valve disease or rheumatic heart disease
		I10-I15	Hypertensive disease
		I20-I25	Ischemic heart disease
		I26-I28	Pulmonary heart disease and pulmonary vascular disease
		I30-I52	Other forms of heart disease
		I60-I69	Cerebrovascular disease
		I70-I79	Diseases of arteries, arterioles and capillaries
		I80-I89	Diseases of veins, lymphatic vessels and lymph nodes, not elsewhere classified
		I95-I99	Other and unspecified disorders of the circulatory system

Supplemental Table 2. Description of ATC order codes from claims

Medication	ATC code	Description
Statin	C10AA	HMG CoA reductase inhibitors
	C10BA	HMG CoA reductase inhibitors in combination with other lipid modifying agents
	C10BX	HMG CoA reductase inhibitors, other combinations
Other lipid-lowering drugs, excluding statins	C10 (Excluding C10AA, C10BA and C10BX)	Lipid modifying agents
Antiplatelet	B01AC06	Acetylsalicylic acid
	B01AC04	Clopidogrel
	B01AC22	Prasugrel
	B01AC05	Ticlopidine1,3,4
	B01AC07	Dipyridamole
	B01AC23	Ccilostazol1,2,4
	B01AC24	Ticagrelor
Anticoagulants	B01AA03	Warfarin
	B01AE07	Dabigatran etexilate
	B01AF01	Rivaroxaban
	B01AF02	Apixaban
NSAIDs	M01A	Antiinflammatory and antirheumatic products, non-steroids
ACE inhibitor	C09A	Ace inhibitors, plain
	C09B	Ace inhibitors, combinations
ARB	C09C	Angiotensin II antagonists, plain
	C09D	Angiotensin II antagonists, combinations
Beta blocker	C07	Beta blocking agents
Calcium channel blocker	C08	Calcium channel blockers
	C07FB	Beta blocking agents and calcium channel blockers1,2,4
	C09BB	ACE inhibitors and calcium channel blockers3
	C09DB	Angiotensin II antagonists and calcium channel blockers

Medication	ATC code	Description
Thiazides	C03A	Low-ceiling diuretics, thiazides
Loop diuretics	C03C	High-ceiling diuretics
Metformin	A10BA02	Metformin
Insulin	A10AD	Insulin

Supplemental Table 3. In-hospital and 30-day mortality between TAVR and SAVR after propensity score overlap weighting

	TAVR group		SAVR group	
	No. of event	%	No. of event	%
In-hospital mortality	14	3.83	41	10.92
30-day mortality	8	2.26	30	8.08

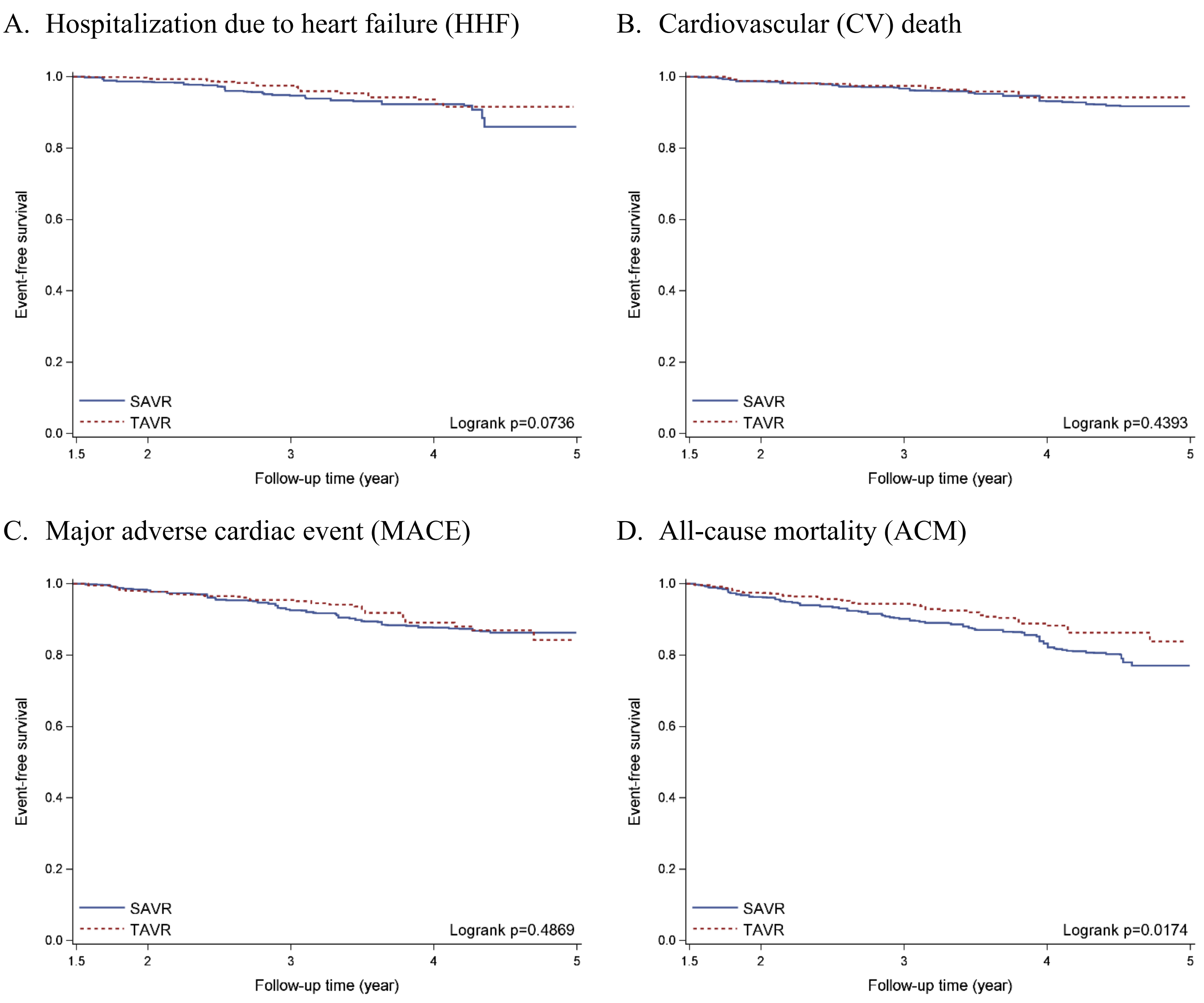

Supplementary Figure 1. Landmark analysis: primary and secondary outcomes from the 18 months after surgery, including Hospitalization due to heart failure (A), Cardiovascular (CV) death (B), C. Major adverse cardiac event (MACE) (C), and all-cause mortality (ACM) (D).
